# Unraveling the Crosstalk between Lipids and NADPH Oxidases in Diabetic Kidney Disease

**DOI:** 10.3390/pharmaceutics15051360

**Published:** 2023-04-28

**Authors:** Rachel Njeim, Sahar Alkhansa, Alessia Fornoni

**Affiliations:** 1 Katz Family Division of Nephrology and Hypertension, Department of Medicine, University of Miami Miller School of Medicine, Miami, FL 33136, USA; 2 Peggy and Harold Katz Family Drug Discovery Center, University of Miami Miller School of Medicine, Miami, FL 33136, USA; 3 Department of Anatomy, Cell Biology and Physiological Sciences, Faculty of Medicine, American University of Beirut, Beirut 1107-2020, Lebanon; 4 AUB Diabetes, American University of Beirut, Beirut 1107-2020, Lebanon

**Keywords:** diabetic kidney disease, lipid dysmetabolism, renal lipids, reactive oxygen species, NADPH oxidases

## Abstract

Diabetic kidney disease (DKD) is a serious complication of diabetes mellitus and a leading cause of end-stage renal disease. Abnormal lipid metabolism and intrarenal accumulation of lipids have been shown to be strongly correlated with the development and progression of diabetic kidney disease (DKD). Cholesterol, phospholipids, triglycerides, fatty acids, and sphingolipids are among the lipids that are altered in DKD, and their renal accumulation has been linked to the pathogenesis of the disease. In addition, NADPH oxidase-induced production of reactive oxygen species (ROS) plays a critical role in the development of DKD. Several types of lipids have been found to be tightly linked to NADPH oxidase-induced ROS production. This review aims to explore the interplay between lipids and NADPH oxidases in order to provide new insights into the pathogenesis of DKD and identify more effective targeted therapies for the disease.

## 1. Introduction

Diabetic kidney disease (DKD), a leading cause of end-stage renal disease (ESRD), affects 20% to 40% of all individuals with diabetes and accounts for significant morbidity and mortality in patients with both type 1 (T1DM) and type 2 (T2DM) diabetes mellitus [1]. DKD is characterized by persistent albuminuria and a relentless decline in the glomerular filtration rate (GFR), both indicating a progressive impairment of renal function [2]. This loss in function is also associated with well-described histological and morphological features including glomerular hypertrophy, mesangial expansion, thickening of the glomerular and tubular basement membranes, glomerulosclerosis with Kimmelstiel–Wilson nodules, and tubulointerstitial fibrosis [2,3,4]. Podocyte injury, a main feature of DKD, is mainly identified by a decrease in the podocyte count, an effacement of foot processes, and a loss of slit diaphragm proteins. These typical phenotypic changes are associated with a defective glomerular filtration barrier and the development of proteinuria. Among these different morphological changes, podocyte loss is considered to be a strong predictor of DKD progression in patients with T1DM and T2DM [5,6,7,8,9]. 

While considerable research has focused on glucose and its metabolism to understand the pathophysiology of diabetes-induced microvascular complications, abnormal lipid metabolism and renal accumulation of lipids have also been suggested to play a key role in the pathogenesis of clinical and experimental DKD [10,11,12,13,14,15]. Lipid accumulation was in fact reported as early as Kimmelstiel and Wilson’s description of DKD [16]. Since then, experimental studies have strongly proposed that examining the altered intracellular lipid metabolism may serve as a mean to discover potential therapeutic targets in patients with DKD [12,14]. 

Moreover, a large body of evidence highlights the overproduction of reactive oxygen species (ROS) as a common denominator link for the major mechanistic pathways involved in the onset and progression of DKD [17]. ROS consist of radical and non-radical oxygen species formed by the partial reduction of oxygen, such as superoxide anion (O_2_^−^), hydrogen peroxide (H_2_O_2_), and hydroxyl radical (HO^•^). Among the different sources of ROS, the reduced nicotinamide adenine dinucleotide phosphate (NADPH) oxidases are of particular importance since their sole biological function is the production of ROS. The NADPH oxidases family comprises Nox1, Nox2, Nox3, Nox4, Nox5, DUOX1, and DUOX2 isoforms [18]. Multiple preclinical and clinical studies have identified NADPH oxidases, specifically Nox4 (previously known as RENOX), as major contributors to the pathophysiology of DKD [19,20,21,22,23,24,25,26]. 

Interestingly, several lines of evidence suggest that lipids play an important role in altering cellular redox homeostasis by regulating NADPH oxidases and impairing the formation of mitochondrial super-complexes [27]. Herein, we aim to review the recent progress in elucidating the interaction between altered lipid metabolism and NADPH oxidases with a primary focus on DKD. We highlight the functional relevance of the crosstalk between lipids, NADPH oxidases, and, more broadly, redox homeostasis in the pathogenesis of DKD. 

## 2. The Role of Renal Lipids in the Pathogenesis of Diabetic Kidney Disease

### 2.1. Cholesterol

Lipids are fundamental building blocks of cells. Balancing lipid uptake, synthesis, utilization, and storage helps regulate cellular lipid homeostasis. While lipids are essential for physiological functions, increasing evidence suggests that altered lipid homeostasis and abnormal lipid accumulation may contribute to injury in non-adipose tissues, ultimately resulting in organ dysfunction [10,14,28,29,30]. Phospholipids, triglycerides, and non-esterified (free of unsaturated) fatty acids (NEFAs) are the major lipid classes present in the kidney. It has been previously shown that an altered renal lipid metabolism favors the net accumulation of cholesterol and triglycerides in the kidney cortex of experimental models of DKD [10,11,14]. Renal lipid accumulation occurs in association with the dysregulation of genes involved in lipid metabolism. Kidney biopsies of patients with DKD showed a significant reduction in genes involved in fatty acid β-oxidation pathways including peroxisome proliferator-activated receptor (PPAR)-alpha, carnitine palmitoyl transferase 1 (CPT1), acyl-CoA oxidase (ACOX), and L-type fatty acid binding protein (L-FABP) [10]. Moreover, the expression of cholesterol uptake receptors including low-density lipoprotein receptors (LDLr), oxidized LDL, and acetylated LDL was significantly elevated, while the expression of genes involved in cholesterol efflux including ATP-binding cassette A1 (ABCA1), ATP-binding cassette G1 (ABCG1), and apolipoprotein E (apoE) was significantly decreased [10]. These data suggest that targeting renal lipid metabolism could be a potential therapy to slow the progression of DKD. 

LDLr and 3-hydroxy-3-methylglutaryl-CoA (HMG-CoA) reductase are involved in maintaining cholesterol uptake and synthesis, respectively, and are both predominantly controlled by sterol regulatory element binding protein-2 (SREBP-2) in the human mesangial cell line [31,32]. SREBP cleavage-activating protein (SCAP) is regarded as the chaperone of SREBP-2 and shuttles SREBP-2 from the endoplasmic reticulum (ER) to the Golgi apparatus for activation by proteolytic cleavage [33]. The cleaved N-terminal fragments of SREBP-2 (nSREBP-2) then activate LDLr and HMG-CoA reductase, resulting in enhanced cholesterol uptake and synthesis. When cells contain sufficient cholesterol, the SCAP-SREBP complex is retained in the ER, thence downregulating LDLr and HMG-CoA reductase expression [34]. A recent study outlined that cholesterol contributed to DKD through the SCAP-SREBP-2 pathway and reported an accumulation of lipid droplets and an increase in HMG-CoA reductase, LDLr, SREBP-2, and SCAP in the kidneys of diabetic rats [35]. Interestingly, inflammation was shown to be another contributor to the increased lipid droplet accumulation in the kidneys of *db*/*db* mice that resulted from elevated protein levels of LDLr, SCAP, and SREBP-2 [36]. Angiotensin II levels were also associated with podocyte injury via elevated expression of LDLr, SREBP-1, SREBP-2, and HMG-CoA reductase and decreased expression of ABCA1 [37]. 

In addition, SREBP-2 has been implicated in the regulation of proprotein convertase subtilisin/kexin type 9 (PCSK9), a key protein in lipid metabolism [38]. PCSK9 is a serine protease enzyme that binds to surface LDL receptors, causing their degradation and subsequently higher plasma LDL-C levels [39,40]. PCSK9 is mainly expressed in the liver and to a lesser extent in the small intestine, kidney, and cerebellum [41,42]. Plasma PCSK9 levels have been shown to be elevated in patients with glomerular filtration barrier disorders such as nephrotic syndrome [43,44]. Moreover, a systematic review concluded that PCSK9 inhibitors are safe, reliable, and effective medications for decreasing LDL cholesterol levels in patients with chronic kidney diseases [45]. Since diabetes is a leading cause of chronic kidney disease, the effect of PCSK9 inhibitor alirocumab was investigated in patients with T1DM and T2DM. Leiter et al. showed that patients with diabetes who were on insulin benefited from receiving the combination of alirocumab and statin or alirocumab alone in patients who could not tolerate statins. After 24 weeks, LDL cholesterol levels were reduced by about 47.8% in patients with Type 1 DM and by 49% in patients with T2DM [46]. However, when it comes to the role of PCSK9 in the pathogenesis of DKD, data are scarce and further investigation is needed. One study showed that in non-dialysis DKD, neither eGFR nor albuminuria influenced plasma PCSK9 levels, but PCSK9 plasma levels were elevated in patients on lipid-lowering therapy [47]. Similar results were reported in non-diabetic patients with chronic kidney disease who were not on dialysis and did not receive statins [48]. However, neither study included a healthy control group. In a study in which healthy subjects were integrated into the analysis, serum PCSK9 levels were approximately doubled in chronic kidney patients compared to the control and were inversely correlated with eGFR [49]. Therefore, more clinical trials and research are needed to investigate the role of PCSK9 in DKD.

ABCA1 is an ATP-dependent transmembrane protein that mediates the efflux of cholesterol and phospholipids to apolipoproteins [50,51]. Loss of ABCA1 function impairs cholesterol efflux and results in cholesterol accumulation in the kidneys [11,52]. Decreased ABCA1 expression has been shown to be positively correlated with markers of DKD progression [27]. Notably, we previously reported increased cholesterol in association with the downregulation of ABCA1 in normal human podocytes exposed to sera from patients with DKD and in glomeruli from patients with early diabetes, while no changes in LDLr and HMG-CoA reductase were observed [14]. Furthermore, we previously showed that elevated tumor necrosis factor (TNF) levels contributed to free cholesterol-dependent podocyte depletion via a reduction in ABCA1-mediated cholesterol efflux and decreased cholesterol esterification by sterol-O-acyltransferase 1 (SOAT1) [53]. Intriguingly, podocyte-specific ABCA1 deficiency aggravated TNF-induced albuminuria, which was fairly prevented by cholesterol depletion with cyclodextrin. In addition, genetic overexpression of ABCA1 or cholesterol depletion was sufficient to attenuate TNF-induced albuminuria and prevent DKD progression in mice with podocyte-specific nuclear factor of activated T cells 1 (NFATc1) activation, a new model of glomerulosclerosis resembling DKD [53]. Moreover, we found that the inhibition of SOAT1 in human podocytes reduced lipotoxicity-mediated podocyte injury in DKD in association with increased ABCA1 expression and ABCA1-mediated cholesterol efflux [54]. Importantly, *db*/*db* mice deficient in SOAT1 showed reduced albuminuria, renal lipid accumulation, foot process effacement, and mesangial expansion [54]. Finally, small-molecule ABCA1 inducers that are currently being tested in phase II clinical trials were found to be protective in experimental DKD [27]. These findings underline the intracellular accumulation of cholesterol as a major mediator of the progression of DKD. 

### 2.2. Fatty Acids and Triglycerides

In addition to cholesterol, fatty acids and triglycerides have also been highlighted as key players in the pathogenesis of DKD [55,56]. We previously described increased triglyceride content in the kidney cortices of podocyte-specific deletion of ABCA1 (Abca1^fl/fl^) mice [27]. In addition, in Akita and OVE26 mice, two genetic models of type 1 diabetes, increased renal triglycerides were correlated with an increased expression of sterol regulatory element-binding protein (SREBP)-1c and carbohydrate response element-binding protein (ChREBP), which, taken together, contributed to an increased fatty acid synthesis [11]. Increased renal triglycerides were also associated with a decreased expression of PPAR-α and PPAR-δ, which led to decreased fatty acid oxidation [11]. Additionally, a decreased expression of farnesoid X receptor (FXR), a negative regulator of SREBP-1c and ChREBP and a positive regulator of PPAR-α in the liver, was also found to be correlated with increased renal triglycerides [11]. Reduced fatty acid oxidation gene expression has also been observed in both Abca1^fl/fl^ and BTBR^ob/ob^ mice. Moreover, human podocytes cultured in the presence of sera from patients with DKD have shown reduced expression of fatty acid oxidation genes [27]. These findings suggest that restoring proper lipid metabolism and increasing fatty acid oxidation locally in the kidneys may both serve as potential therapeutic approaches to preventing and treating DKD.

### 2.3. Sphingolipids

Sphingolipids have also been shown to play a significant role in the development and progression of DKD. Sphingolipids are typically classified as ceramides (CERs), sphingomyelins (SMs), or glycosphingolipids (GSL) along with their metabolites. Ceramide acts as a precursor for other biologically active sphingolipids including sphingosine (SPH), CER-1-phosphate (C1P), and sphingosine-1-phosphate (S1P) [57]. CER, C1P, and S1P have been demonstrated to regulate a variety of cellular processes such as cell proliferation, maturation, differentiation, apoptosis, autophagy, inflammation, immunity, and membrane fluidity. The role of S1P in proper kidney functioning remains poorly elucidated. S1P is derived from SPH by the action of sphingosine kinase-1 (SK1), and it is cleaved by S1P lyase [58]. Previous reports showed that neutral ceramidase, SK activity, and S1P levels were all significantly increased in isolated glomeruli from rats treated with streptozotocin (STZ) for 4 days, suggesting a possible involvement of S1P in the glomerular proliferative response in the early-stage of DKD [59]. In addition, SK1 was found to be associated with enhanced levels of the matrix constituent fibronectin in STZ-induced diabetic rat kidneys and glomerular mesangial cells exposed to high glucose [60]. In line with this notion, renal S1P levels were also significantly elevated in STZ-induced diabetic mice [61]. In mice, knockout of the Sgpl1 gene that encodes S1P lyase 1 contributed to foot process effacement and severe proteinuria [62]. Interestingly, other studies have proposed that, contrary to expectations, the upregulation of SK-1 could actually be reno-protective since higher albuminuria and increased connective tissue growth factor (CTGF) expression were found in kidney sections of SK-1 knockout mice as compared to wild-type C57BL/6 mice [63]. Moreover, the administration of S1P1 receptor agonists in experimental DKD models has been reported to attenuate albuminuria and renal dysfunction [64]. In patients with type 2 diabetes, plasma levels of S1P were decreased and showed a significant decline in parallel to kidney dysfunction, suggesting that severe nephropathy may have led to the loss in albumin-associated S1P [65]. Further studies on S1P remain necessary to uncover its exact role in the development and progression of DKD. 

As mentioned, ceramide is a biologically active sphingolipid that serves as a substrate for the production of C1P and S1P [58]. Ceramide can be synthesized de novo, generated by hydrolysis from SM by sphingomyelinases, or generated by the breakdown of GSL and galactosylceramide to dihydroceramide with subsequent hydrolyzation [66]. Increased levels of ceramide have been found in the plasma of patients with diabetes and were strongly correlated with the severity of insulin resistance [67]. In addition, increased ceramide production due to upregulated expression of serine palmitoyl transferase (SPT), a key enzyme involved in ceramide de novo synthesis, was reported in tubular epithelial cells and microvascular endothelial cells and was correlated with increased apoptosis in STZ-induced DKD [68]. Sphingomyelin phosphodiesterase acid-like 3b (SMPDL3b) is a lipid raft enzyme that regulates integrin activation, cell migration, and cell survival in podocytes [69,70]. We previously described SMPDL3b overexpression in normal human podocytes exposed to sera from patients with DKD and in glomeruli of patients with insulin resistance and DKD [70]. In our recent study, SMPDL3b overexpression was found to negatively affect the availability of C1P in human podocytes in vitro and in kidney cortices of *db*/*db* mice in vivo [71]. More importantly, we found that human podocytes that overexpressed SMPDL3b were unable to phosphorylate protein kinase B (PKB), also known as Akt, in response to insulin stimulation, indicating insulin resistance. Podocyte-specific Smpdl3b deficiency in diabetic mice restored C1P levels and protected them from DKD. Furthermore, exogenous administration of C1P restored insulin signaling in vitro and prevented DKD progression in vivo [71]. Collectively, these findings highlight SMPDL3b as a modulator of insulin signaling and suggest that C1P may represent a lipid therapeutic strategy to treat DKD. 

A potential role for glycosphingolipid accumulation in DKD was raised in an earlier work showing that hyperglycemia was associated with enhanced synthesis of glucosylceramide and ganglioside GM3, ultimately leading to renal hypertrophy in STZ-induced diabetic rats [72]. The increased glucosylceramide synthesis was paralleled with an increase in UDP-glucose concentration and reducing equivalents in the form of NADPH [72]. Therefore, inhibiting the de novo synthesis of glycosphingolipids may represent a therapeutic modality for DKD. Glycosphingolipid synthesis may be targeted with potent and selective inhibitors of glucosylceramide synthase [73,74]. In experimental models of diabetic kidney disease, the inhibition of glucosylceramide synthase attenuated fibrosis, decreased extracellular matrix proteins, and reversed mesangial cell hypertrophy by reducing hyperglycemia-induced phosphorylation of SMAD3 and Akt [75].

## 3. The Role of NADPH Oxidases in the Pathogenesis of Diabetic Kidney Disease

ROS are thought to be the final common denominator linking the different pathogenic mechanisms of diabetic vascular complications and specifically of DKD [17]. Excessive ROS production mediates renal fibrosis and tissue inflammation, imposing deleterious consequences on the structure and function of the kidney [76]. Among the different sources of ROS, NADPH oxidases appear to play a key pathophysiological role in DKD [77,78]. Recent studies identified NADPH oxidases as major sources of ROS in the glomeruli and kidney cortices of rats with T1DM [22,23,24]. More specifically, Nox4-induced ROS production was found to mediate renal hypertrophy and increase fibronectin expression, contributing to the progression of DKD [22]. Consistently, Eid A. et al. previously showed that high glucose induced apoptosis of cultured mouse podocytes through the production of ROS via sequential upregulation of cytochrome P450 of the 4A family (CYP4A) and the two major Nox isoforms (Nox1 and Nox4). It was reported that 20-hydroxyeicosatetraenoic acid (20-HETE), a major product of CYP4A, increased NADPH-dependent ROS production, upregulated Nox1 and Nox4 protein expression, and mediated podocyte apoptosis. To further confirm the results, inhibition of CYP4A prevented oxidative stress and podocyte apoptosis in vitro and attenuated albuminuria and podocyte loss in OVE26 mice [25]. Moreover, it was recently demonstrated that podocyte loss induced by either type 1 diabetes in vivo or by exposure to high glucose concentrations in vitro was mediated by the activation of the mechanistic target of rapamycin (mTOR) complex 1 (mTORC1) through the inactivation of the AMP-activated protein kinase (AMPK)/tuberin pathway. Notably, mTORC1 activation was correlated with enhanced oxidative stress and increased Nox1 and Nox4 protein expression. Inhibition of mTORC1 using rapamycin significantly attenuated podocyte depletion and glomerular injury by inhibiting Nox4-dependent ROS generation [19]. Furthermore, a recent study suggested a novel function of mTOR complex 2 (mTORC2) in NADPH oxidase-dependent ROS production and podocyte apoptosis that mediates disruption of podocyte integrity and albuminuria in type 1 diabetes [20]. Taken together, these results suggest that mTOR inhibition and/or NADPH oxidase inhibition may constitute therapeutic approaches to DKD. A recent study provided key findings that underlined the possible role of Nox5-mediated ROS production in the pathogenesis and progression of DKD [26]. In human kidney biopsies, Nox5 was found to be expressed in mesangial cells of glomeruli and appeared to be upregulated in diabetes. In addition, vascular smooth muscle cell/mesangial cell-specific overexpression of Nox5 in a mouse model of diabetic nephropathy worsened diabetes-induced albuminuria, renal hypertrophy, mesangial expansion, ECM accumulation, glomerulosclerosis, and renal inflammation via increased ROS generation [26]. Collectively, these findings highlight the central role of excessive NADPH oxidase-derived ROS in the kidney in precipitating renal injury in diabetes. 

## 4. The Crosstalk between Lipids and NADPH Oxidases in Diabetic Kidney Disease

### 4.1. Cholesterol, Phospholipids, and NADPH Oxidase Signaling in DKD

Dysfunction of cholesterol and phospholipid metabolism has been shown to be associated with an alteration in the NADPH oxidase signaling pathway and an increase in oxidative stress, resulting in DKD. One of the well-described morphological features of DKD is hypertrophy. However, the underlying complex signaling pathways that regulate renal hypertrophy remain poorly investigated. Increasing evidence has highlighted calcineurin (Cn), a calcium- and calmodulin-dependent phosphatase, as a mediator of hypertrophy in DKD [79,80,81,82,83,84]. Cn, which is activated by insulin-like growth factor-1 (IGF-1) and transforming growth factor (TGF)-β, was shown to mediate hypertrophy in renal mesangial cells; its inhibition with cyclosporin or tacrolimus, however, impeded both glomerular and whole-kidney hypertrophy in diabetic rats [79,85]. More specifically, the calcineurin A-β (CnAβ) subunit appeared to mediate the renal hypertrophy observed in DKD [83]. In addition, CnAβ was shown to regulate NFAT, which in turn plays a crucial role in the pathogenesis of DKD [86]. It is also well documented that NADPH oxidases (specifically Nox4) mediate hypertrophy and fibronectin expression in DKD [22]. The expected increase in ROS in response to high glucose was shown to be significantly attenuated in primary kidney cells lacking a catalytic subunit of Cn (CnAβ^−/−^), suggesting that Cn acts upstream of Nox [87]. Consistently, loss of CnAβ decreased high-glucose-induced Nox2 and Nox4 expression. Moreover, inhibition of NFAT decreased Nox2 and Nox4 expression, while its overexpression upregulated Nox2 and Nox4 expression [87]. These results suggest that the CnAβ/NFAT pathway regulates NADPH oxidases, thus contributing to the renal hypertrophy observed in diabetes. More importantly, NFAT activation has been shown to suppress ABCA1 expression [53]. ABCA1 loss-of-function mutations are typically observed in patients with Tangier’s disease, which is characterized by the presence of foamy podocytes on kidney biopsies [88]. Intriguingly, these patients only develop minimal proteinuria, suggesting that loss of ABCA1 function may cause lipid accumulation but is insufficient to result in glomerular injury [88]. In fact, neither the knockdown of ABCA1 in podocytes (siABCA1) in vitro nor the podocyte-specific deletion of ABCA1 in vivo was enough to cause podocyte apoptosis or glomerular injury, respectively [53]. Notably, fibroblasts from patients with Tangier’s disease are susceptible to a significant accumulation of cardiolipin [89], a mitochondrial-specific phospholipid that is crucial in maintaining mitochondrial function and integrity [90]. This suggests that ABCA1 may play a role in mediating the mitochondrial dysfunction seen in DKD [91,92]. Indeed, in our recent study we observed that ABCA1 knockdown in podocytes resulted in reduced oxygen consumption as well as in changes in the oxidative phosphorylation (OXPHOS) complexes with cardiolipin accumulation [27]. Moreover, in vivo podocyte-specific deletion of ABCA1 (Abca1^fl/fl^) increased the susceptibility of mice to DKD [27]. To further establish the role of cardiolipin in DKD and its link with ABCA1, cardiolipin peroxidation was inhibited using elamipretide, which attenuated DKD in vivo and inhibited ABCA1-dependent podocyte injury both in vitro and in vivo. Remarkably, ABCA1 knockdown in podocytes was associated with an increase in superoxide dismutase 2 (SOD2), a mitochondrial ROS scavenger [27]. This may provide a plausible explanation as to why ABCA1 deficiency alone is not sufficient to cause glomerular injury. When challenged in a diabetic state in which SOD2 is reduced, ABCA1 deficiency resulted in exacerbated podocyte injury [27]. These observations demonstrate a pathway correlating ABCA1 deficiency to cardiolipin-driven mitochondrial dysfunction, ultimately leading to DKD. Furthermore, loss of ABCA1 expression and function can act as a susceptibility factor for DKD, and its pharmacological induction with cyclodextrin or its genetic overexpression was shown to prevent the progression of DKD [14,53]. Collectively, these observations highlight a potential interplay between ABCA1 and NADPH oxidases while taking into account that NFAT not only activates NADPH oxidases but also suppresses ABCA1 expression. They also reveal that loss of ABCA1 expression is associated with an alteration in the OXPHOS complexes with cardiolipin-driven mitochondrial dysfunction, resulting in a shift in redox homeostasis in the kidney. 

Of note, some anti-hyperlipidemic drugs that act by lowering hypercholesterolemia have been shown to exert an antioxidant effect by inhibiting the NADPH oxidase pathway (Table 1). Statins act by inhibiting HMG-CoA reductase, a major enzyme in cholesterol synthesis, thus decreasing endogenous cholesterol production [93]. The reno-protective role of statins in kidney injury and DKD has been investigated in several clinical trials [94,95], but the molecular mechanisms underlying this protective role have yet to be understood. A recent study showed that treatment with pitavastatin ameliorated nephropathy in *db*/*db* mice by downregulating Nox4 expression in the kidney [96]. Similarly, atorvastatin attenuated diabetes-associated renal injury by reducing Nox4-induced ROS production and RhoA activity and by normalizing Akt/glycogen synthase kinase 3 beta (GSK3β) signaling pathways, which are known to be important players in renal pathology [97]. Likewise, atorvastatin treatment reduced high-fat diet (HFD)-induced upregulation of Nox2 and Nox4 mRNA and protein expression in collecting ducts of rats with 5/6 nephrectomy and a high-fat diet [98]. Other plausible explanations for the reno-protective role of statins include decreased lipid peroxidation, elevated antioxidant levels, decreased accumulation of advanced glycosylation end products, and maintenance of podocyte integrity [35,99,100,101]. These observations suggest that statins exert their reno-protective role independently of their systemic lipid-lowering effects, indicating that it is the tissue-specific hypercholesterolemia rather than the systemic one that plays a role in the pathogenesis of DKD. Probucol is another anti-hyperlipidemic drug that has recently been shown to have an antioxidant effect. *Db/db* mice fed a probucol-containing diet (1.0%) showed reduced levels of cholesterol, triglyceride, and LDL compared to *db*/*db* mice fed a regular diet [102]. Importantly, treatment with probucol ameliorated DKD as assessed by a decrease in UAE and glomerular injury and an increase in podocyte number. This reno-protective effect of probucol might be attributed to the decrease in oxidative stress observed in the kidneys of *db*/*db* mice treated with probucol as measured by oxidative stress markers including thiobarbituric acid reactive substances (TBARS). Although Nox4 and Nox2 expressions were both elevated in the kidneys of untreated *db*/*db* mice, probucol treatment significantly attenuated only the expression of Nox2 [102]. Both TBARS (r = 0.92; *p* < 0.01) and cholesterol (r = 0.79; *p* < 0.01) were correlated with albuminuria [102], highlighting the importance of targeting the lipid dysmetabolism–oxidative stress axis in DKD. 

PCSK9 inhibition has emerged as a novel target for decreasing LDL cholesterol and preventing coronary heart disease. Treatments aimed at inhibiting PCSK9 are continuously being developed. Drugs targeting PCSK9 directly (such as the FDA-approved monoclonal antibody evolocumab) or affecting its expression (such as Ginkgolide B) have been shown to be effective in the case of kidney diseases. However, the mechanism by which PCSK9 inhibition might exert its protective effect in kidney disease is not well elucidated [45,103]. A possible mechanism by which PCSK9 inhibition might exert its protective effect is by decreasing oxidative stress [104]. Ginkgolide B treatment has been shown to inhibit Nox4 expression and attenuate mitochondrial ROS generation [105]. On the other hand, fenofibrate, a peroxisome proliferator-activated receptor-α (PPAR-α) agonist, is another FDA-approved drug to treat patients with primary hypercholesterolemia, hypertriglyceridemia, or mixed dyslipidemia [106]. Fenofibrate has been shown to improve renal function by inhibiting Nox4 and reducing oxidative damage. It also inhibits apoptosis and attenuates inflammation in STZ-induced diabetic rats [107]. In addition, drugs that induce cholesterol efflux, such as cyclodextrin or ezetimibe, demonstrated a reno-protective effect in DKD and DKD-like glomerulosclerosis [14,27]. Interestingly, both cyclodextrin and ezetimibe have been shown to play a role in attenuating oxidative stress [108,109]. Niacin has been also shown to enhance lipid metabolism and have anti-inflammatory and antioxidant effects. Treating nephrectomized rats with niacin ameliorated kidney injury and was able to attenuate the upregulation of NOX-4, p22^phox^, and p47^phox^ [110]. 

Aside from the interplay between cholesterol and NADPH oxidases, an interaction between phospholipids and NADPH oxidases has also been shown to play a key role in the progression of DKD. Lysophosphatidic acid (LPA) is a small phospholipid derivative present in most tissues and body fluids. LPA acts as a potent mitogen by activating the six G protein-coupled LPA receptors (LPAR1 to LPAR6). LPA is associated with a wide range of biological cell responses such as proliferation, migration, and apoptosis [111,112]. It can also induce cell damage through the generation of ROS, inflammatory cytokines, and pro-fibrotic factors in several tissues including the kidney [111,112,113,114,115]. Increasing evidence has reported elevated LPA levels in the sera of patients with diabetes as well as in the kidney cortex of *db*/*db* mice, suggesting a possible role of LPA in DKD [116,117]. Consistently, a recent study showed that LPA signaling was activated in the STZ-induced mouse model of type 1 diabetes as manifested by a significant increase in the levels of LPA type 1 receptor (LPAR1) mRNA and protein as compared to a non-diabetic model [118]. Treatment with AM095, a specific pharmacological inhibitor of LPAR1, attenuated glomerular injury in the kidney of STZ-induced diabetic mice [118]. To investigate the potential role of oxidative stress in LPA signaling in diabetic kidney failure, ROS production was measured in LPA-treated SV40 MES13 cells, an immortalized mouse mesangial cell line, using flow cytometry. LPA treatment was shown to increase ROS production, whereas treatment with AM095 significantly inhibited this increase. Interestingly, increased ROS production was positively correlated with the NADPH oxidase protein expression level. Treatment with AM095 attenuated this increase in mesangial cells as well as in the kidneys of diabetic mice [118]. Toll-like receptor 4 (TLR4) activates the inflammatory response and simultaneously activates NADPH oxidases. Treatment with AM095 inhibited LPA-induced TLR4 expression in mesangial cells and in the kidneys of STZ-induced diabetic mice. In addition, AM095 suppressed LPA-induced fibrotic factors and pro-inflammatory cytokines through the downregulation of phosphorylated NFκBp65 and c-Jun N-terminal kinases (JNK) [118]. Pharmacological or siRNA inhibition of TLR4 and NADPH oxidase mimicked the effects of AM095 [118]. These findings suggest that the LPAR1-specific antagonist AM095 attenuates glomerular injury in type 1 diabetic model by inhibiting TLR4/NF-κB and the NADPH oxidase system, consequently suppressing the inflammatory signaling cascade in the kidneys of diabetic mice. More importantly, this study highlighted a potential crosstalk between LPA and NADPH oxidases in the pathogenesis of DKD.

### 4.2. Fatty Acids, Triglycerides, and NADPH Oxidase Signaling in DKD

Accumulating evidence has elucidated a possible crosstalk between triglycerides, fatty acids, and NADPH oxidases in DKD. SREBP-1 is an important transcription factor regulating lipid synthesis. The potential involvement of SREBP-1 in kidney diseases has been well elucidated in DKD experimental models such as Akita and OVE26 mouse models of T1D [11,13,15,119]. SREBP-1c is preferentially active in driving the transcription of genes involved in fatty acid synthesis [120]. SREBP-1c activation results in the accumulation of triglycerides in the kidney and is associated with mesangial matrix expansion, increased expression of profibrotic factors, and proteinuria [11,13,15,119]. In line with the notion that accumulation of tissue lipids may be a potential source for oxidative stress, a recent study showed that transgenic mice overexpressing nuclear (active) SREBP-1c under control of the phosphoenolpyruvate carboxykinase (PEPCK) promoter (PEPCK-TgSREBP-1c) exhibited renal abnormalities resembling DKD [121]. SREBP-1c-overexpressing glomeruli also had a markedly higher expression of components of the NADPH oxidase system (including p47^phox^ and p67^phox^) as compared to control mice. This indicated that glomerular SREBP-1c could directly induce oxidative stress through NADPH oxidases. More importantly, similar observations were reported in STZ-induced diabetic mice with activation of endogenous SREBP-1c. Interestingly, renal injury and oxidative stress were attenuated in SREBP-1-null mice. In addition, adenoviral overexpression of nuclear SREBP-1c in MES13 mesangial cells increased TGF-β and p47^phox^ expression, whereas adenoviral dominant-negative SREBP-1c suppressed TGF-β expression [121]. These findings suggest that activation of glomerular SREBP-1c could contribute to the development of DKD through a NADPH oxidase-dependent mechanism. Surprisingly, a recent study showed that inhibition of SREBP-1 by fatostatin did not improve DKD in T1D mice [122]. Therefore, further investigation of the efficacy of SREBP inhibitors and the specific role of SREBP-1 in the pathogenesis of DKD is warranted. 

PPAR-α is a ligand-dependent nuclear receptor that plays a crucial role in lipid metabolism [123]. PPAR-α can be activated by fatty acids, prostaglandins, and exogenous compounds such as fibrates [123]. PPAR-α is primarily expressed in tissues with high fatty acid metabolism activity and helps regulate genes involved in fatty acid synthesis and oxidation [124]. PPAR-α has been reported to be expressed in glomerular and renal tubular cells with the role of regulating lipid accumulation in the kidney [125]. In fact, fenofibrate, a PPAR-α agonist, was found to exert reno-protective effects in DKD by attenuating lipotoxicity in the kidney [126,127]. PPAR-α deficiency has been shown to worsen the progression of DKD by increasing extracellular matrix production and inflammation in the kidney [128]. However, the underlying molecular mechanisms of the reno-protective effects of PPAR-α agonists remain poorly elucidated. It was recently demonstrated that diabetic PPAR-α^−/−^ mice had a significant increase in Nox4 expression as compared to diabetic wild-type mice, suggesting that the reno-protective role of PPAR-α agonists may be attributed to their antioxidant activity [129]. Additionally, treatment with fenofibrate improved renal function in diabetic rats by attenuating oxidative stress and decreasing Nox4 expression [107]. A recent study revealed that K-877 (pemafibrate), a selective PPAR-α modulator (SPPARMα), significantly attenuated albuminuria in *db*/*db* mice as compared to their control littermates. It also induced a significant decrease in total diacylglycerol (DAG) content in the glomeruli of *db*/*db* mice [130]. Among multiple sources, protein kinase C (PKC)-induced activation of NADPH oxidases has been described as a major source for ROS production in diabetes mellitus [131,132]. PKC activation in hyperglycemia is likely to be correlated with elevated levels of free fatty acids (FFAs) and accumulation of lipid intermediates such as DAG [133]. Inhibition of the DAG-PKC-NADPH oxidase pathway provides a promising therapeutic approach for DKD. K-877 was shown to attenuate mesangial expansion in *db*/*db* mice by inhibiting the DAG-PKC-NADPH oxidase pathway, ultimately leading to a decrease in oxidative stress [130]. Taken together, these studies underline a crucial interplay between PPAR-α, DAG, PKC, and NADPH oxidases. 

FXR is another important nuclear receptor that has been shown to play a pivotal role in regulating both bile acid and lipid metabolism [134,135,136]. FXR is activated by endogenous bile acids, with chenodeoxycholic acid being the most potent endogenous ligand followed by deoxycholic acid, lithocholic acid, and lastly cholic acid [137]. FXR is abundantly expressed in the liver, intestine, kidney, and adrenal glands, but to a lesser extent in heart and adipose tissues [138,139,140]. In mouse kidneys, FXR was shown to be expressed in both isolated proximal tubule cells and glomeruli. Additionally, FXR has been detected in both cultured mouse mesangial cells and podocytes [141]. In kidney biopsies from patients with nephropathy associated with diabetes and obesity, FXR mRNA is significantly decreased in both glomeruli and tubules [142]. FXR knockout mice with STZ-induced type 1 diabetes demonstrated an exacerbated renal injury compared to age-matched C57BL/6 wild-type mice injected with STZ. These mice exhibited an increase in albuminuria, basement membrane thickening, glomerulosclerosis, podocyte injury, mesangial expansion, and tubulointerstitial fibrosis [143]. Importantly, FXR deficiency augmented neutral lipid accumulation in both glomeruli and tubulointerstitium and increased renal cholesterol and triglyceride levels [143]. Renal lipid dysmetabolism was accompanied by an increase in the expression of SREBP-1c and its target genes acetyl-CoA carboxylase (ACC), fatty acid synthase (FAS), and stearoyl-CoA desaturase-1 (SCD-1) [143], which play a role in fatty acid and triglyceride synthesis. Moreover, FXR deficiency led to the upregulation of the LDL receptor and lectin-like oxidized LDL receptor-1 (LOX-1), increasing cholesterol and oxidized LDL uptake [143]. Remarkably, treatment with FXR agonists ameliorated DKD in different mice models of diabetic nephropathy including *db*/*db* mice with type 2 diabetes [141]; DBA/2J mice fed a high-fat, high-cholesterol diet [12]; and STZ-induced type 1 diabetic DBA/2J mice [143]. Activating FXR restored renal lipid homeostasis by inhibiting ChREBP and SREBP-1c, reducing fatty acid synthesis and triglyceride accumulation [12,141,143]. Furthermore, the FXR agonist INT-747 increased the expression of genes involved in fatty acid oxidation and lipid catabolism, including PPAR-α, PPAR-γ coactivator-1α (PGC-1α), CPT1a, uncoupling protein-2 (UCP-2), and lipoprotein lipase (LPL) [12]. In addition, FXR activation attenuated oxidative stress as assessed by a decrease in Nox2 and p22^phox^ expression [12,141,143]. 

**Figure 1:**
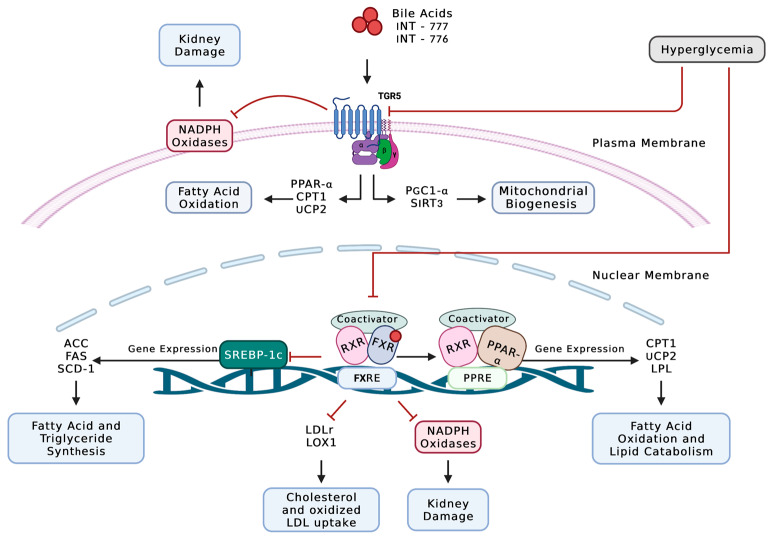
Diabetes-induced alteration in fatty acid and triglyceride metabolism upregulates NADPH oxidase-dependent ROS production in the kidney. TGR5 is bile acid-activated GPCR that plays a pivotal role in upregulating mitochondrial biogenesis and fatty acid β-oxidation. FXR is a bile acid-activated nuclear receptor that plays an important role in regulating lipid metabolism. FXR activation upregulates the expression of PPAR-α, which is involved in lipid catabolism and fatty acid β-oxidation, while inhibiting the expression of genes that are involved in cholesterol and oxidized LDL-uptake. In addition, FXR activation inhibits the expression of SREPB-1c and its target genes, which are involved in the synthesis of fatty acids and triglycerides. In DKD, both receptors are downregulated, leading to lipid accumulation and injury. Importantly, the decrease in TGR5 and FXR expression leads to an increase in NADPH oxidase-dependent ROS production, further exacerbating kidney injury. ACC: acetyl-CoA carboxylase; CPT1: carnitine palmitoyltransferase 1; FAS: fatty acid synthase; FXR: farnesoid X receptor; FXRE: FXR response element; LOX-1: lectin-like oxidized LDL receptor-1; LDLr: low-density lipoprotein receptors; LPL: lipoprotein lipase; PPAR-α: peroxisome proliferator-activated receptor- α; PPRE: PPAR response element; PGC-1α: peroxisome proliferator-activated receptor-gamma coactivator 1 alpha; RXR: retinoid X receptor; SCD-1: stearoyl-CoA desaturase-1; SIRT3: NAD-dependent deacetylase sirtuin-3, mitochondrial; SREBP1-c: sterol regulatory element-binding protein 1-c; TGR5: G-protein-coupled bile acid receptor; UCP-2: uncoupling protein-2.

Besides FXR, bile acids also activate the membrane-bound G protein-coupled receptor (TGR5). Although both are activated by bile acids, TGR5 expression and function are distinct from FXR except in some cases in which their roles complement [144,145]. TGR5 mRNA expression was detected in the small intestine, gall bladder, liver, brown adipose tissue, spleen, placenta, lung, kidneys, and some areas of the nervous system [144,145,146]. In the kidneys, TGR5 mRNA expression was detected in both human and mouse tubules and glomeruli [147]. Notably, TGR5 mRNA expression in kidney biopsy specimens from patients with DKD or obesity-related glomerulopathy was significantly decreased compared to normal kidney biopsy specimens, and TGR5 mRNA levels were inversely correlated with the progression of the disease [147]. Treatment of diabetic *db*/*db* mice with the selective TGR5 agonist INT-777 ameliorated diabetic renal injury. INT-777 attenuated urinary albumin excretion, podocyte loss and injury, mesangial expansion, and macrophage infiltration [147]. INT-777 is thought to exert its reno-protective effect through upregulating the expression of master regulators of mitochondrial biogenesis (such as SIRT3 and PGC-1α), inhibitors of oxidative stress (such as Nrf-1), as well as inducers of fatty acid β-oxidation (such as PPAR-α, CPT1β, and UCP-2) [147]. Interestingly, targeting the FXR/TGR5 pathway with the dual agonist INT-767 attenuated renal injury by preventing lipid accumulation, enhancing mitochondrial biogenesis and fatty acid β-oxidation, and decreasing oxidative stress [142]. Particularly, treatment with INT-767 reduced Nox2 and p22-phlox mRNA expression [142]. Collectively, these data suggest a possible interplay between FXR/TGR5 pathway, lipid metabolism, and Nox-dependent oxidative stress (Figure 1). 

### 4.3. Sphingolipids and NADPH Oxidase Signaling in DKD

Increasing evidence has described an interplay between ceramide and redox signaling that modulates various cell functions and leads to the development of renal dysfunction and cardiovascular disease. It was previously shown that ceramide stimulated the activation of ROS-producing enzymes including xanthine oxidase, NO synthase, the mitochondrial respiratory chain enzymes, and NADPH oxidases [148,149]. In particular, ceramide has been shown to activate NADPH oxidases in a variety of mammalian cell types such as human aortic smooth muscle cells, endothelial cells (ECs), and macrophages [150,151,152]. The activation of NADPH oxidases requires the translocation and aggregation of its subunits. Therefore, it was suggested that ceramide induces the activation of NADPH oxidases by mediating the fusion of small raft domains to ceramide-enriched membrane platforms, subsequently clustering the subunits of NADPH oxidases and leading to an active enzymatic complex. In fact, the activation of the sphingomyelinase enzyme mediates the production of ceramide molecules, which spontaneously assemble to form microdomains that fuse to large ceramide-enriched membrane platforms [153]. In such membrane raft platforms, NADPH oxidase subunits assemble into an active enzymatic complex that produces ROS [154,155]. The ceramide-enriched membrane platform with Nox subunit clustering and enzyme activation is termed the membrane raft redox signaling platform (MRRSP) [57]. The MRRSP is dependent on the activation of sphingomyelinase to produce ceramide in response to various physiological or pathological stimuli [57]. MRRSP and its associated oxidative stress through increased NADPH oxidase activity have been reported to mediate glomerular injury and sclerosis during hyperhomocysteinemia (hHcys) [156]. 

Homocysteine (Hcys), a sulfur-containing amino acid, is formed as a metabolic intermediate during the metabolism of methionine. Increasing evidence has highlighted the role of Hcys in health and disease [157]. Perturbations in the intracellular metabolism of homocysteine and its subsequent circulating accumulation, termed hyperhomocysteinemia (hHcys), have been well documented in the progression of chronic kidney disease (CKD) [157,158]. In addition, in T2DM, hHcys was shown to be strongly correlated with an increased risk of cardiovascular disease (CVD) and mortality [159]. In previous experimental studies, Hcys also appeared to increase the production of extracellular matrix (ECM), consequently promoting the sclerotic process in vessel walls and other tissues [160,161]. Moreover, it was previously demonstrated that sustained elevations of plasma Hcys mediated glomerulosclerosis in both a hypertensive Dahl salt-sensitive rat model and a normotensive rat model [162]. In a hospital-based case-control association study, elevated Hcys plasma levels were causally associated with an increased risk of DKD in Chinese patients with diabetes [163]. Recent studies have indicated that Hcys increases superoxide production via NADPH oxidases, subsequently stimulating the production of tissue inhibitor of metalloproteinase-1 (TIMP-1) in renal mesangial cells and leading to collagen deposition [164]. Considering that ceramide is a major activator of NADPH oxidases in different cells [57,165,166] and that NADPH oxidases contain the Rac protein, a regulatory component that allows for a high affinity binding to lipids, a recent study investigated the role of ceramide in the Hcys-induced activation of NADPH oxidases in rat mesangial cells. Hcys was shown to increase de novo production of ceramide in rat mesangial cells, and it markedly increased the level of GTP-bound Rac, which was paralleled by enhanced activity of NADPH oxidases. Treatment with a Rac GTPase inhibitor (GDPbS) and a de novo ceramide synthesis inhibitor (fumonisin B1 (FB1)) abolished Hcys or ceramide-mediated effects [156]. These results indicate that Hcys activates NADPH oxidases in a ceramide-dependent pathway. In vivo, treatment with the de novo ceramide synthesis inhibitor myriocin decreased renal ceramide levels and NADPH oxidase activity and attenuated glomerular injury in uninephrectomized Sprague Dawley rats fed a folate-free diet for 8 weeks. In rats treated with apocynin, a NADPH oxidase inhibitor, similar beneficial results were seen in protecting the glomeruli from hHcys-induced injury [167]. These observations support the notion that de novo ceramide production is involved in Hcys-induced NADPH oxidase activity in the kidney and further underline the interplay between sphingolipids and NADPH oxidases in DKD. 

Notably, the formation of lipid raft (LR) redox signaling platforms in glomerular endothelial cells (GEnCs) accounts for the early event of hHcys-induced glomerular injury. A recent study revealed a co-localization of LR clusters with the NADPH oxidase subunits gp91^phox^ and p47^phox^ in GEnC membranes in response to hHcys. Moreover, the NADPH oxidase activity was significantly increased upon Hcys stimulation. Interestingly, hHcys resulted in increased permeability of GEnC with disruption of the microtubule network, leading to glomerular injury [168]. In fact, endothelial cell injury leads to an increase in glomerular capillary permeability, enhancing the efflux of albumin from glomerular capillaries. This induces mesangial cell expansion, leading to eventual glomerular injury and sclerosis [169]. 

Acid sphingomyelinase (Asm), a ceramide-producing enzyme, was also shown to play a key role in mediating glomerulosclerosis and podocyte injury associated with increased NADPH oxidase activity during hHcys [170]. Renal ceramide production, Asm mRNA and activity, urinary albumin excretion, the glomerular damage index (GDI), NADPH-induced ROS production in the renal cortex, and podocyte injury were all attenuated in uninephrectomized Asm-knockout mice (Asm^−/−^) or Asm short hairpin RNA (shRNA)-transfected wild-type mice compared with wild-type (Asm*^+/+^*) mice [170]. Furthermore, the hHcys-induced glomerular injury was significantly improved in gp91^phox^ knockout (gp91^−/−^) mice. Proteinuria, the glomerular damage index (GDI), foot process effacement, and podocyte loss due to hHcys were all significantly attenuated in gp91^−/−^ mice as compared to wild-type (gp91^+/+^) mice, further establishing the role of NADPH oxidases in hHcys-induced podocyte injury [171].

Recently, MRRSP-induced ROS generation was shown to induce the activation of NOD-like receptor family, pyrin domain containing 3 (NLRP3) inflammasome. NLRP3 mediates the inflammatory response in different cells including renal podocytes and tubular cells [172]. The redox activation of NLRP3 inflammasome is considered an underlying pathogenic mechanism of glomerulosclerosis and consequent ESRD [173,174]. More importantly, NLRP3 activation mediates the production of caspase-1, interleukin (IL)-1β, IL-18, and other cytokines, thus promoting the inflammatory cascade reaction, which is crucial for the pathogenesis of DKD [175,176]. Glomerular injury associated with obesity has also been shown to be attributed to the MRRSPs in GEnCs. Visfatin, an adipokine reported in obesity and diabetes mellitus, was shown to increase sphingomyelinase activity, leading to an increased production of ceramide and clustering of NADPH oxidase subunits gp91^phox^ and p47^phox^ in membrane rafts, forming MRRSP. The formation of MRRSP was inhibited by prior treatment with MR disruptor filipin, sphingomyelinase inhibitor amitriptyline, sphingomyelinase siRNA, gp91^phox^ siRNA, and adiponectin. Visfatin also increased the permeability of GEnC in culture and resulted in the disruption of microtubular networks [177]. 

Recent studies have reported that S1P, another biologically active sphingolipid, can stimulate Nox2 and Nox4-dependent ROS production in different cells. S1P has been shown to increase Ca2+ sensitization in vascular smooth muscle cells (VSMCs) by acting on Rho A/Rho kinase pathway, subsequently activating NADPH oxidase-induced ROS production [57]. Mechanistically, S1P was found to mediate p47^phox^ translocation in fibroblasts, thus leading to Nox activation [178]. In the kidney, S1P is considered to be a potent vasoconstrictor of the preglomerular microvasculature, which is closely correlated with the pathogenesis of DKD [179]. The interplay between S1P and NADPH oxidases in the kidney warrants further investigation. The activation of NADPH oxidases by sphingolipids such as ceramide and S1P and the decrease in the activity of antioxidant defenses such as nitric oxide (NO) and SOD are described as crucial players in the crosstalk between sphingolipids and ROS production in DKD [75,180]. 

## 5. A Two-Way Crosstalk: Increased ROS Production May Cause Lipid Dysmetabolism

Increased ROS generation in DKD may also lead to lipid dysmetabolism. Several studies associated the development of obesity and metabolic syndrome with NADPH oxidase-induced ROS production. Nox4 activity was shown to increase transiently during obesity development in control mice fed a high fat, high sucrose (HFHS) diet for 8 to 16 weeks [181]. Adipocyte-specific deletion of Nox4 delayed the onset of insulin resistance in mice fed with the HFHS diet, improved plasma lipids, and attenuated inflammation in both liver and adipose tissue [181]. Moreover, fat accumulation and BMI were positively correlated with systemic oxidative stress in both humans and mice [182,183]. Interestingly, treatment of KKAy mice, a model of diabetic obesity, with the NADPH oxidase inhibitor apocynin reduced the production of ROS in adipose tissue and attenuated adipocytokine dysregulation. In addition, treatment with apocynin attenuated insulin, glucose, plasma triglycerides, and hepatic triglycerides levels in KKAy mice [182]. Intriguingly, mice overexpressing the Nox subunit p22^phox^ in smooth muscle cells (tg^sm/p22phox^) developed an evident increase in body weight, insulin resistance, leptin resistance, and other metabolic syndrome characteristics upon high-fat feeding [184]. Collectively, these data suggest that NADPH oxidases may play a role in lipid metabolism. 

**Figure 2:**
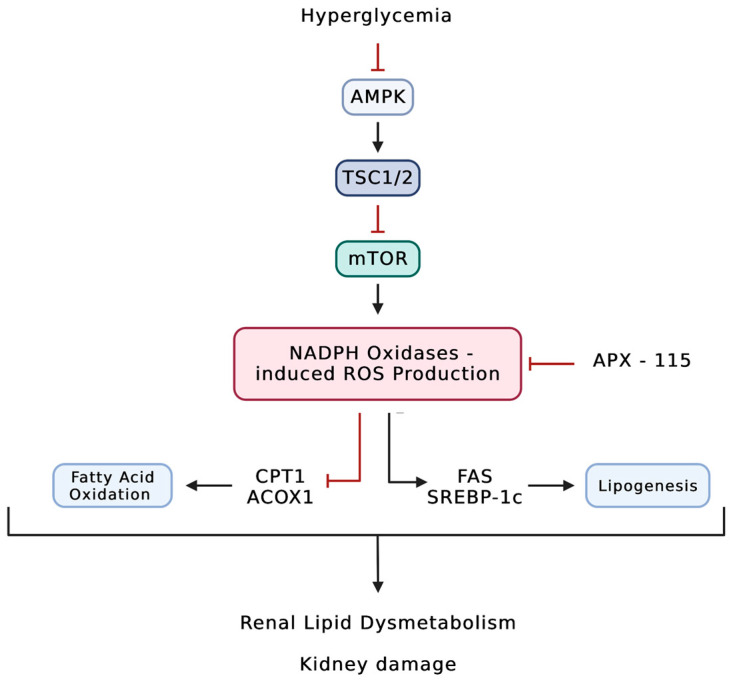
Diabetes induces upregulation of NADPH oxidase-dependent ROS production, leading to renal lipid dysmetabolism. In DKD, diabetes inhibits the AMPK pathway, causing upregulation of the mTOR pathway. Subsequently, mTOR activation leads to the activation of NADPH oxidases. NADPH oxidase-induced ROS production has been shown to alter renal lipid metabolism by inhibiting fatty acid β-oxidation and upregulating lipogenesis. ACOX1: acyl-CoA oxidase 1; AMPK; 5’ AMP-activated protein kinase; CPT1: carnitine palmitoyltransferase-1; FAS: fatty acid synthase; mTOR: mechanistic target of rapamycin; SREBP-1c: sterol regulatory element-binding protein 1-c; TSC1: hamartin; TSC2: tuberin.

In addition, Grove et al. previously reported that the inhibition of ROS production with pyridoxamine inhibited the accumulation of phospholipid and glycolipid species from four different classes including gangliosides, sulfoglycosphingolipids, lysophospholipids, and phosphatidylethanolamines in the glomeruli and/or tubules of endothelial nitric oxide synthase deficient C57BLKS *db*/*db* mice. Furthermore, the inhibition of ROS production prevented nephropathy progression in these mice [116]. Thus, increased ROS generation in DKD could promote the synthesis of GSLs. Importantly, inhibiting NADPH oxidases using the first-in-class pan-NADPH oxidase APX-115 was shown to protect *db*/*db* mice from renal injury by reducing cholesterol and triglyceride content locally in the kidneys [185]. Similarly, in STZ-induced mice, treatment with APX-115 was able to reverse diabetes-induced lipid accumulation in the kidney. APX-115 was able to restore the expression of the lipolytic enzymes such as CPT1 and ACOX1 that are downregulated in diabetes, suggesting a role of NADPH oxidases in fatty acid β-oxidation in both mitochondria and peroxisomes. Furthermore, treatment with APX-115 was able to decrease the expression of the lipogenic enzymes fatty acid synthase and SREBP-1c (Figure 2) [186]. In a study conducted by Lee et al., mice that expressed human Nox5 in podocytes (NOX5 pod^+^) were fed an HFD. The body weight of NOX5 pod^+^ mice did not differ significantly from their control littermates. However, treatment with APX-115 but not ACT-705500 (ACT), which is a Nox1 and Nox4 inhibitor, reduced body weight gain in NOX5 pod^+^ mice that were fed an HFD. Additionally, APX-115 treatment decreased total cholesterol and total triglyceride levels and inhibited lipid accumulation in the liver, suggesting that APX-115 improved HFD-induced lipid dysmetabolism [187]. Notably, APX-115 treatment also ameliorated diabetic kidney disease in NOX5 pod^+^ mice [187]. These data suggest that NADPH oxidases play an important role in regulating both systemic and local lipid metabolism. However, further studies should be conducted to elucidate the mechanisms behind Nox-induced lipid dysmetabolism.

## 6. Conclusions

Renal accumulation of lipids and abnormal intracellular lipid metabolism play a central role in the pathogenesis of diabetic kidney disease. Examining lipids and their altered metabolism has thus been of great interest to scientists in the race to find a treatment for DKD. The accumulation of cholesterol in the kidneys appears to be a major mediator of DKD and is favored by the dysregulation of several lipid metabolism genes. Fatty acids, triglycerides, and sphingolipids also play a key role in the pathogenesis of DKD. Along with lipid accumulation and altered metabolism, the production of ROS by NADPH oxidases is also recognized as a major contributor to the pathophysiology of DKD. Inhibiting NADPH oxidase appears to be a plausible approach to treat DKD. Here, we summarized the clinical and experimental evidence supporting the central role of lipids in the pathogenesis of DKD with a particular focus on the lipids that also alter redox homeostasis in an NADPH oxidase-dependent manner. We believe that further studies on the role of cholesterol, phospholipids, triglycerides, fatty acids, and sphingolipids in activating NADPH oxidases should show great promise in finding potential therapies for DKD. 

## Figures and Tables

**Table 1:** The Effect of Anti-Hyperlipidemic Drugs on Regulating the NADPH Oxidase Pathway.

Anti-Hyperlipidemic Drugs	Mechanism of Action	Effect on Oxidative Stress and NADPH Oxidases	Major Findings	Reference
Pitavastatin	Inhibits HMG-CoA reductase	- Decreases Nox4	- Decreases albuminuria - Decreases urinary 8-OHdG and 8-epi-PGF2α - Normalizes renal mesangial expansion - Decreases TGF-β1 and fibronectin expression	[96]
Atorvastatin	Inhibits HMG-CoA reductase	- Decreases Nox4	- Decreases albuminuria - Reduces renal hypertrophy - Reduces ROS generation - Reduces RhoA activity - Normalizes Akt/glycogen synthase kinase 3 beta (GSK3β) signaling pathways	[97,98]
Probucol	Increases the rate of LDL catabolism	- Decreases Nox2	- Decreases UAE - Improves fibrosis - Increases podocyte number - Decreases ROS production	[102]
Evolocumab	Monoclonal antibody against PCSK9	- Decreases intracellular H_2_O_2_ production in peripheral blood mononuclear cells (PBMCs)	- Lowers plasma lipids - Improves arterial stiffness	[104]
Ginkgolide B	Downregulates PCSK-9 expression	- Decreases Nox4 along with the attenuation of mitochondrial ROS generation	- Alleviates the Ox-LDL-induced inflammatory cascades and altered lipid metabolism in human umbilical vein endothelial cells (HUVECs)	[105]
Fenofibrate	Peroxisome proliferator-activated receptor-α (PPAR-α) agonist	- Potentiates antioxidant defense systems by enhancing catalase and superoxide dismutase enzyme activities and glutathione content - Reduces oxidative damage by lowering malondialdehyde (MDA) generation - Attenuates the expression of Nox4	- Improves creatinine clearance and protein excretion - Lowers plasma levels of blood urea nitrogen, creatinine, and uric acid	[107]
Cyclodextrin	Removes cholesterol from cells	- Reduced the production of ROS following ingestion of apoptotic cells in both WT and Abca1^−/−^Abcg1^−/−^ efferocytes	- Preserves viability of macrophages following exposure to oxidized phospholipids and/or apoptotic cells	[108]
Ezetimibe	Inhibits of Niemann–Pick C1-like 1 protein	- Decreases the urinary excretion of 8-hydroxy-2’-deoxyguanosine, a parameter of oxidative stress, and increases the urinary excretion of nitrate and nitrite (NOx)	- Reduces urinary albumin excretion	[109]
Niacin	Lipid-lowering drug	- Lowers plasma malondialdehyde (MDA) - Reverses or markedly attenuates the upregulation of NOX-4, p22^phox^, and p47^phox^ - Does not affect gp91^phox^ expression	- Ameliorates hypertension, proteinuria, glomerulosclerosis, and tubulointerstitial injury - Reduces histological injury and mitigates upregulation of oxidative and inflammatory systems in the remnant kidney	[110]

## Data Availability

Not applicable.
